# Physical Activity in Mental Health Treatment: Clinician Perspectives and Practices

**DOI:** 10.3390/clinpract15070129

**Published:** 2025-07-08

**Authors:** Madeline Crichton, Barbara Fenesi

**Affiliations:** Faculty of Education, Western University, London, ON N6G 1G7, Canada; mcricht3@uwo.ca

**Keywords:** physical activity, mental health, psychotherapy, clinician perspectives

## Abstract

**Background/Objectives**: The beneficial effects of physical activity on mental health and well-being are well established. The integration of physical activity into psychotherapeutic treatment for mental health difficulty holds promise as an avenue to reduce symptoms and support well-being. Mental health clinicians have previously indicated an interest in the use of physical activity in treatment, but it is unclear to what extent physical activity interventions are implemented in clinical mental health care. The present study aimed to understand mental health clinicians’ practices related to physical activity, as well as to investigate their related training and knowledge. **Methods**: Semi-structured interviews were conducted with mental health clinicians, including registered psychologists, psychotherapists, and social workers. Inductive content analysis was performed to identify key themes related to practices, training experiences, and training interests. **Results**: Clinicians reported making recommendations for physical activity and using a range of in-session strategies to include physical activity in mental health treatment. Clinicians reported that their knowledge and training about physical activity was obtained primarily from informal sources. Clinicians indicated an interest in further training, with an emphasis on practical strategies. **Conclusions**: Mental health clinicians demonstrated an interest in the use of physical activity as part of psychotherapeutic treatment. Some clinicians routinely integrate physical activity into treatment, while others express a need for further training in this area.

## 1. Introduction

The mental health benefits of physical activity are well established [1,2,3,4]. This is in addition to the positive effects of physical activity on other health outcomes, such as cardiovascular health and lowered rates of obesity [5,6,7]. Despite the multi-faceted benefits of engaging in regular physical activity, only a small minority of children are engaging in sufficient physical activity based on World Health Organization (WHO) guidelines [8,9,10]. In Canada, the majority of children and adolescents are sedentary for more than half of their waking hours [11]. The mental health burden among children and adolescents is significant globally [12,13] and the current service availability is not sufficient to meet these needs [14,15]. In recent years, the effect of the COVID-19 pandemic has further increased the need for youth mental health services [16,17]. Greater promotion of physical activity among children and youth holds potential as a tool to support mental health and well-being at individual and population levels [18,19]. However, despite the growing evidence supporting the role of physical activity in mental health, and the recommendation of physical activity as a first-line treatment for depression [20], it is unclear to what extent physical activity-based treatments and strategies are being implemented. As such, the current study aims to investigate the beliefs, practices, and knowledge of mental health clinicians related to the use of physical activity in their current practice. The perspectives of clinicians are valuable to consider as they play a direct, active role in service delivery and may serve as gatekeepers for which services are available to individuals seeking mental health care.

Physical activity has been identified as beneficial for a range of mental health conditions and neurodevelopmental disorders that are commonly treated with psychotherapy, including depression, anxiety, and attention-deficit/hyperactivity disorder (ADHD) [21,22,23]. Physical activity is also supportive of general mental well-being and contributing factors such as self-esteem and resilience, even in the absence of a specific mental disorder [1,24]. Children and adolescents who engage in more physical activity have a lower likelihood of experiencing depression or other mood disorders, as well as improved executive functioning and cognitive performance compared to their less active peers [25,26]. There are several proposed explanations for the link between physical activity and improved mental health. There is evidence supportive of neurobiological factors, such as greater synaptic plasticity, increased blood flow to the brain, increased expression of Brain-Derived Neurotropic Factor (BDNF), and greater availability of neurotransmitters implicated in mood and anxiety disorders [27,28,29]. Psychosocial factors may also contribute, given that physical activity often leads to opportunities for social connection, skill development and mastery, and access to nature, all of which have been found to improve well-being, self-confidence, and self-esteem [30,31,32].

The inclusion of physical activity as part of mental health treatment can be considered through the lens of the biopsychosocial model. The biopsychosocial model, first introduced by George Engel in 1977 as an alternative to the biomedical model, asserts that health, including mental health, is a product of biological, social, and psychological factors [33]. Physical activity works on all levels of the model, impacting biological, social, and psychological health [34]. The psychological determinants of mental health have traditionally been the targets for psychotherapeutic intervention, with some emphasis on social factors [35], although there has been an increase in clinician interest in taking a whole-person approach that includes biological factors [36]. Often the biological contributors to mental health are addressed through medication treatments, outside the purview of most practitioners of psychotherapy. However, the inclusion of physical activity as a component of mental health treatment may provide an opportunity to address the biological contributors to mental health in the context of non-medical mental health care and psychotherapy.

The integration of physical activity, or recommendations to engage in physical activity, into clinical practice holds potential as an avenue for these interventions to be accessible for individuals experiencing mental health difficulty. Indeed, a significant majority of mental health clinicians are aware of the benefits of physical activity for mental health [37,38,39], and many clinicians regularly discuss physical activity with their clients [40,41,42]. The view that physical activity is an appropriate and desirable component of mental health intervention has increased in prevalence over time [37,40,43]. Yet many clinicians remain uncomfortable recommending physical activity or feel that they do not have adequate knowledge or training to engage in this practice [44,45]. Over 60% of clinicians report wanting additional training about how to use physical activity in their clinical practice [37,42,46]. Clinicians want training on topics such as motivating clients to participate in physical activity, what type of physical activity is best, how to assess suitability for physical activity, and how to help clients access physical activity resources [46]. In addition to being desirable to clinicians, formal training in physical activity promotion leads to higher rates of providing physical activity advice and counselling [47].

Although there are gaps in training and knowledge, many clinicians do report regular discussion of physical activity with clients. However, details about these discussions are lacking in the existing literature, such as frameworks or techniques utilized by clinicians and which specific populations, symptom presentation, or phase of treatment are deemed best suited by clinicians for physical activity interventions and whether direct methods of physical activity integration are being used by clinicians. As such, the current study employs a qualitative approach with the aim of eliciting greater specificity about clinical methods. Additionally, the existing literature has largely focused on adult client populations. There may be different methods and techniques used in the treatment of children and adolescents compared to treatment for adults. For example, there may be a role for parents and guardians in the treatment process, and physical activity modalities may be different (e.g., physical education class or sports participation in children compared to attending workout classes or going to the gym in adults). Understanding the nuances of how clinicians currently employ physical activity in their practice, particularly when working with children and youth, is necessary to determining the next steps in promoting the use of physical activity-based interventions among clinical providers, as well as determining the most effective strategies that can be employed by clinicians to deliver physical activity interventions to their child and adolescent clients.

Therefore, the current study used a qualitative approach to investigate the methods and strategies used by clinicians to integrate physical activity into mental health care, as well as to understand what training clinicians have received in this area and what further training they wish to receive. It was hypothesized that clinicians use multiple approaches, including discussion and providing recommendations, as well as direct, in-session physical activity integration. Clinician training was expected to be variable and to include both formal and informal sources of knowledge and training. Clinicians were expected to be seeking further training to expand their knowledge base about the benefits and risks of physical activity, as well as practical strategies as resources, consistent with previously described training interests [38,46].

## 2. Materials and Methods

### 2.1. Participants

Participants were fourteen mental health clinicians registered to provide services in the province of Ontario. The participants included seven registered psychotherapists, 5 registered psychologists, and 2 registered social workers. Eight participants had been registered in their profession for less than 5 years and six participants had been registered for more than 5 years. There were twelve female and two male participants. Eleven participants worked in private practice, one worked in a hospital setting, and two worked in both private practice and hospital settings. Participants were recruited through professional networks, including listservs and social media platforms. Participants were self-selected based on interest.

### 2.2. Procedure

Individual, semi-structured interviews were completed by each participant via Zoom video conferencing. Prior to engaging in the interviews, eligibility was confirmed and participants provided informed and voluntary consent. The interviews lasted approximately 30 min and the audio was recorded for transcription. Interviews were completed between May 2023 and July 2023. Participants were compensated for their time in the form of a CAD 10 gift card. The study received approval from the institution’s research ethics board.

### 2.3. Materials

The interview format was semi-structured. Four primary interview questions (Appendix A) were prepared by the research team to elicit responses that would address the research questions described above. The questions were developed for the present study and were face-valid for their relevance in addressing the research questions. The interview questions were posed to each participant verbatim by the researcher, and follow-up questions were asked flexibly based on participant responses to gather more detailed information.

### 2.4. Qualitative Data Analysis

The data collection and analysis were centered within a post-positivist paradigm, which acknowledges that researchers will necessarily hold background knowledge and biases which may impact their observations and interpretations of data [48]. Given that both the primary researcher and the research assistant involved in coding were graduate trainees in clinical mental health programs whose training experiences, clinical frameworks, and background knowledge could impact their perceptions of the data, the post-positivist paradigm was viewed as most appropriate.

Interview responses were analyzed using inductive content analysis. The content analysis procedure involves compressing text into categories based on specific content codes and themes [49,50]. It is inductive in nature, since the content codes are derived from the data. This procedure was used to identify recurring themes in the interview responses and report this information. This allowed for trends and common responses to be identified across participants.

The interviews were transcribed using Zoom’s built-in transcription service and then manually checked for accuracy against the audio recordings. Two researchers read the transcripts to become familiar with the data. The same two researchers then developed a preliminary codebook that categorized the data based on initial, emerging themes in the interviews. The research team consulted on the codebook and discussed the extent to which it captured key themes. A final codebook was developed. This codebook was reapplied to the transcripts using MAXQDA (Version 24), a software program for qualitative analysis. The frequency of each theme was determined. The researchers independently reviewed and coded each transcript. Any discrepancies in the coding were resolved through discussion until consensus was reached.

## 3. Results

The data are presented separately for each research question, with each section divided by themes and subthemes identified in the data. The themes and subthemes are presented in order of frequency, determined by the number of times participants discussed each theme in the interviews. Illustrative quotations are provided from the interview transcripts.

### 3.1. Research Question 1: How Do Mental Health Clinicians Include Physical Activity as Part of Psychotherapy for Their Child and Adolescent Clients?

The first research question aimed to identify the strategies and processes used by clinicians when including physical activity in psychotherapy for children and adolescents. Table 1 provides a frequency summary of the themes that were expressed under Research Question 1.

#### 3.1.1. Theme 1: Making Physical Activity Recommendations (Frequency 171)

The most common way that clinicians identified integrating physical activity into their clinical practice was by making recommendations. This theme captured discussion of making physical activity recommendations in general, as well as strategies mentioned by clinicians for making their recommendations more effectively. Subthemes included individualizing physical activity recommendations (which included individualization related to presenting issues, and collaborative problem solving), recommending a mindful approach, making recommendations through a behavioural activation framework, and making efforts to facilitate physical activity outside of the therapy environment.

##### Subtheme 1a: Individualizing Physical Activity Recommendations (Frequency 98)

Clinicians identified that a component of making recommendations was ensuring that recommendations were individualized to the client’s needs. For example, one clinician reported, “I might talk about how, depending on what their interests are and what their presenting concern is and what they are motivated to want to change, I’ll take from those areas and present it to them where they can make small changes and start to see gains.” (Participant 7). Additional trends became apparent within this theme and further sub-subthemes were created, including individualizing based on the presenting issue and collaboratively problem solving an individual approach with a client.

##### Sub-Subtheme 1a: Presenting Issues (Frequency 41)

Some clinicians mentioned specific populations and presenting issues for which they would be more or less likely to recommend physical activity. For example, eating disorders were frequently discussed as an area of concern for which the approach to physical activity would need to be individualized: “Where it can be problematic is with our eating disorder population … activity and fitness is not advised depending on where they’re at in their recovery.” (Participant 4). Conversely, clinicians reported that they were more likely to recommend physical activity for depression or low mood compared to other areas of concern. “I probably recommend physical activity the most, where it pops into my head most quickly, is when I’m talking to a child or adolescent struggling with low mood.” (Participant 2). The role of physical activity in the management of ADHD and behaviour problems was also highlighted by several clinicians: “I emphasize it for a lot of the kids that come to me for behavioral problems, as an emotion regulation, anger outlet to expel some of that energy, as well as for kids with ADHD.” (Participant 4).

##### Sub-Subtheme 1b: Collaborative Problem Solving (Frequency 32)

Several clinicians reported that they aim to be collaborative with their clients when discussing physical activity to ensure that recommendations are workable for the client. As one clinician said, “Let’s be honest, it’s hard to get into a rigid routine if it’s not something that you enjoy … so can we find a way that that’s fun and more engaging?” (Participant 8). Clinicians also reported working with their clients around practical concerns: “If they’re not allowed to go outside or if they live in a high-rise apartment building, then do they have access to a computer? Can we pop on a YouTube video? Do they like to dance?” (Participant 6).

##### Subtheme 1b: Mindful Approach (Frequency 28)

Some clinicians described integrating mindfulness in their approach to recommending physical activity. This included recommending engaging in mindfulness while completing physical activity, as well as taking a mindful approach to reflecting on how they feel while being active. For example, “We incorporated a yoga practice. More of a mindfulness and meditation rather than fitness, but it is still stretching and movement.” (Participant 14). Another clinician reported, “I would bring it up in terms of helping them understand how moving their bodies can change the way they feel.” (Participant 6).

##### Subtheme 1c: Behavioural Activation (Frequency 18)

Clinicians mentioned using the framework of behavioural activation when making recommendations for physical activity as part of treatment for depression and low mood. One clinician described “using a lot of behavioral activation for people with depression. So needing to do something and committing to doing that in order to change the mood.” (Participant 9). Another clinician reported, “In terms of the behavioral activation piece, we’re often talking about ways of getting up and out of their house. Whether that’s going for a walk or taking their dog out or getting them involved in sports.” (Participant 3).

##### Subtheme 1d: Facilitating Physical Activity Outside of Practice (Frequency 9)

A smaller number of clinicians reported working actively to facilitate opportunities for their clients to be physically active outside of the therapy practice. One clinician described facilitating a program that included, “a depression group for teens, 16 to 19, where we would do regular CBT one day and then we would come together a second day of the week to do a one-hour workout class.” (Participant 1). Another clinician mentioned, “trying to work with their school a little bit so that if they’re feeling very restless or overwhelmed then trying to get them out for like a quick walk, even down the hallway.” (Participant 4).

#### 3.1.2. Theme 2: Psychoeducation (Frequency 36)

Another way that clinicians identified utilizing physical activity in their practice was through psychoeducation. Clinicians reported educating their clients about the potential mental health benefits of physical activity during the treatment process. This theme captures psychoeducation in general, as well as the sub-theme of framing the discussion of physical activity in specific ways.

When discussing providing psychoeducation to their clients, clinicians reported: “We’ll provide the education on why physical activity is helpful, and I go through the psycho-ed part of that with them.” (Participant 8) and “I also talk a little bit about how there’s been literature on how regular physical exercise can be as beneficial as pharmaceutical interventions for depression … when I talk about it with teens, it’s just a little bit less technical.” (Participant 7).

##### Subtheme 2a: Framing Physical Activity (Frequency 17)

Clinicians also discussed the specific ways they tend to frame the discussion of physical activity with their clients, for example, to de-emphasize the connection between physical activity and body weight or shape, or to distinguish between daily movement and fitness training. As one clinician described it, “I think it’s really important that when we encourage physical activity, that it’s not from a perspective to stay a certain weight or to achieve a certain goal. It’s focused on ‘how do you feel?’ and that should be the main thing.” (Participant 5).

#### 3.1.3. Theme 3: In-Session Integration of Physical Activity (Frequency 16)

Some clinicians also reported integrating physical activity directly into their sessions with clients. This occurred in a variety of ways. For example, one clinician said “If I’m running a CBT group, in the nicer weather, I’ve taken them outside. We’re talking through CBT strategies, but we’re using little basketball games.” (Participant 8). Another clinician reported, “Our practice certainly encourages exercise during sessions … I actually just got back from a three and a half kilometer walk outside with a kid through the session … we do any type of individual therapy, but moving.” (Participant 3). Other clinicians described the use of physical activity as an exposure to the physical sensations associated with anxiety: “So let’s say they’re experiencing anxiety and they can feel their increased heart rate. Usually, I work with them on practicing strategic ways to increase the heart rate through physical activity.”, and using active play in play therapy modalities: “Sometimes [play therapy] can include … I have this tunnel they can crawl through. We have those big yoga balls that they can jump around on if they want to if they’re a kid that has a little bit more energy and benefits from that.”

#### 3.1.4. Theme 4: Asking About Physical Activity During Assessment or Intake (Frequency 9)

Several clinicians reported including a discussion of physical activity during the intake or initial assessment phase of therapy. “It starts right at the beginning, when I do the initial intake interview with parents or with adolescents, if they’re old enough. That is one of the questions I ask about, what kinds of physical activity they may be getting on average per week, and what that entails, if it’s something that they enjoy, that kind of thing.” (Participant 7).

#### 3.1.5. Theme 5: Physical Activity as an Alternative/Addition to Medication (Frequency 4)

Although our sample did not include professionals with prescription privileges, a few clinicians did discuss the role of medication in treatment and the use of physical activity to augment or replace medication. One clinician said, “I’m often talking about, and some of these kids may be on [medication], for instance, and physicians now think it’s the first line before [medication] to prescribe physical activity for kids.” (Participant 2).

### 3.2. Research Question 2: How Do Clinicians Learn About Physical Activity and Mental Health?

The second research question investigated how clinicians learn about physical activity and mental health, as part of formal training or elsewhere. Table 2 provides a frequency summary of the themes that were expressed under Research Question 2.

#### 3.2.1. Theme 1: Personal Physical Activity Experience (Frequency 18)

Several clinicians reported that their understanding of the role of physical activity in supporting mental health arose through experiences with physical activity in their personal lives. One clinician described “being a lifelong athlete, as I grew and got older, I recognized the positive benefits it was having on my mental health.” (Participant 12). Another reported, “I was doing a kickboxing class myself at Goodlife and I just noticed that although I wasn’t particularly down, I would always be a bit high after the class … and I thought, I have to share this with kids because if it’s doing this for me maybe it will for somebody else too.” (Participant 1).

#### 3.2.2. Theme 2: Research or the Literature (Frequency 16)

Clinicians also reported consulting research and scientific literature to inform their understanding of the role of physical activity and mental health. One clinician reported “reviewing the literature around physical activity and public health.” (Participant 12). Another described their understanding of the literature: “I think there’s been lots of really good evidence talking about exercise and movement, certainly in the depression literature.” (Participant 3).

#### 3.2.3. Theme 3: Lack of Formal Education (Frequency 14)

Interestingly, when asked about the source of their knowledge about physical activity, clinicians frequently discussed not learning about it during their graduate training. Several clinicians mentioned their graduate training specifically: “That was not discussed in my clinical training. Supervisors never talked about it in my clinical training outside of the classroom either.” (Participant 7); “[My university]’s program definitely did not talk about that. That’s not on their list.” (Participant 3); “I went to grad school a very long time ago, but I don’t think this was a hot topic. I don’t think this was particularly covered in any of my courses.” (Participant 2). As another clinician reported, “I don’t know if it was ever specifically part of my training … it almost feels like an idea that we all are supposed to sort of know.” (Participant 5).

#### 3.2.4. Theme 4: Formal Training Outside of Graduate Program (Frequency 8)

A smaller number of clinicians reported having pursued their own formal training in physical activity and related topics, outside of their clinical or graduate training. One clinician described “taking workshops” (Participant 5). Another reported “I’ve tried taking courses on physical fitness and nutrition.” (Participant 4). Others had previous educational experiences, such as an undergraduate degree in kinesiology or training as a yoga instructor, that informed their knowledge of physical activity and mental health.

#### 3.2.5. Theme 5: Clinical Experience (Frequency 5)

Some clinicians also reported that their knowledge of physical activity and mental health emerged through clinical experience. Clinicians reported: “A little bit of learning from clients.” (Participant 4); “One of my clients said physical activity was useful … they felt they were doing something good for themselves and also managing anxiety.” (Participant 13). “It’s just something that a lot of people respond to.” (Participant 10).

#### 3.2.6. Theme 6: Consultation with Colleagues (Frequency 3)

A few clinicians also reported learning about physical activity through consulting with colleagues. One described their experience “talking to different practitioners, talking to people who are trained in different approaches psychologically.” (Participant 5). Another reported “talking to some sports psychologists in the U.S. about what they were doing in terms of incorporating some physical activity.” (Participant 1).

### 3.3. Research Question 3: What More Do Clinicians Want to Know About Physical Activity and Mental Health?

The third research question investigated what additional information or training clinicians would like to receive related to physical activity and mental health. Table 3 provides a frequency summary of the themes that were expressed under Research Question 3.

#### 3.3.1. Theme 1: Resources for Recommending Physical Activity (Frequency 35)

The most frequently identified future need was resources for recommending physical activity. Clinicians expressed an interest in having access to more resources they could use when recommending physical activity or discussing it with their clients. This theme captured an interest in having access to a summary of the research related to physical activity and mental health with clinicians as the intended audience, as well as having access to similar resources with clients as the intended audience. This theme also captured an interest in resources for identifying specific physical activity ideas that could be shared with clients.

##### Subtheme 1a: Research Summary for Clinicians (Frequency 15)

Several clinicians expressed an interest in learning more about the evidence-base for the relationship between physical activity and mental health. As one clinician said, “For my own purpose, I’d love to know the more scientific stuff behind it of how it actually has been proven in studies to affect the brain, and then how to apply it.” (Participant 10). Another clinician said, “If there is any solid evidence floating around that what people are doing is more helpful or unhelpful … if there’s a way of tying it in that seems to actually increase uptake or change things outside of the individual session that you’re doing, that information would be helpful.” (Participant 3).

##### Subtheme 1b: Research Summary for Clients (Frequency 13)

Several clinicians also expressed an interest in accessing materials that could be shared with clients. One clinician reported wanting access to “materials that you can give to children and youth so that they can see. Or things that I could give to parents to help them understand why this is going to be part of the treatment plan.” (Participant 6). Another clinician said they would like to learn “how to help clients understand the benefit for them of doing physical activity, maybe especially for those who aren’t already engaged in physical activity, people who have never had it as a part of their daily framework.” (Participant 9).

##### Subtheme 1c: Physical Activity Ideas (Frequency 5)

Some clinicians also expressed interest in a resource bank of physical activity ideas that they could use when discussing physical activity and helping clients integrate it into their routines. One clinician reported wanting “a resource for clinicians for brainstorming ideas … just because I like kickboxing doesn’t mean that everyone out there is going to like that … having just a giant pool of ideas of how kids can get a bit of cardio, just a huge brainstorm of thoughts would be great.” (Participant 1). Similarly, another clinician said they would like a resource “with different ideas of what they could do. Maybe even something like one of those spinning wheel things with the needle on it, so you could spin and try something new each session.” (Participant 7).

#### 3.3.2. Theme 2: How to Overcome Barriers (Frequency 7)

Several clinicians also reported that they would benefit from additional resources and training in how to support clients to overcome barriers to physical activity participation. “Maybe, some tips about getting around those barriers, whether practical or more social-emotional, like the low mood by virtue interfering with the engagement in physical activity. I would be interested in being more immersed in that and learning more about that.” (Participant 2). Another participant hoped further training could address the question of: “How do you get people there? To follow through with [being physically active]?” (Participant 8).

## 4. Discussion

The present study aimed to understand the methods and strategies employed by mental health clinicians to integrate physical activity into their practice, as well as to understand their training experiences and support needs. As hypothesized, clinicians reported utilizing a variety of in-session activities as well as making recommendations for physical activity to support mental health. They also reported a range of primarily informal training experiences and an interest in accessing further training and resources. The following section will discuss the themes identified in the data and considerations for future research and practice.

### 4.1. How Do Mental Health Clinicians Include Physical Activity as Part of Psychotherapy for Their Child and Adolescent Clients?

#### 4.1.1. Making Recommendations

Making recommendations was the most commonly cited method for integrating physical activity into clinical practice. This is consistent with findings that discussion and recommendations are a primary modality for communicating about physical activity with clients [40,41] Clinicians described several ways in which they make recommendations, such as individualizing recommendations based on presenting issues and client needs or preferences, utilizing mindfulness in recommendations, recommending physical activity as part of behavioural activation, and working to facilitate clients’ physical activity outside of their clinical practice. Clinicians may be limited, depending on practice setting, to talking about physical activity with clients rather than engaging them directly in physical activity. Clinical training and theoretical modalities are also unlikely to include physical activity as a direct feature. As such, the high frequency of making recommendations was expected.

Within this theme, many clinicians emphasized the importance of individualizing physical activity recommendations, based on presenting issues or client circumstances. The importance of being collaborative and understanding barriers that clients face to being active was also repeatedly discussed. This perspective is likely rooted in professional standards and clinical training that recognizes the importance of client participation and collaborative approaches [51,52]. This approach is also consistent with the guidelines for family physicians prescribing physical activity for mental health. Guidelines suggests that recommended daily physical activity be used as a benchmark (e.g., Health Canada recommendation of 60 min of moderate to vigorous physical activity per day for children and adolescents) but that specific goals should be discussed with the patient to ensure feasibility [53]. Clinicians in this study seem to take a similar approach in which the individual needs and goals of the client are prioritized in making physical activity recommendations. Recognizing potential barriers, including emotional barriers, to engagement with physical activity and working to address these may also be beneficial. Although not focused on physical activity, recent work suggests that emotional variables like guilt may play a significant role in health behavior regulation and engagement in therapeutic activities [54].

Clinicians also frequently highlighted presenting issues or diagnoses as a factor impacting their recommendations regarding physical activity. For example, many clinicians reported that they were more likely to recommend physical activity in clients presenting with symptoms of depression. This is consistent with the literature [30,55,56] and indicates that clinicians are aware of the positive impact of physical activity on mood. Conversely, clinicians reported using more caution or limiting recommendations of physical activity in clients presenting with eating disorder symptoms. This is consistent with guidelines suggesting a gradual re-introduction of exercise for underweight individuals and encouraging the social and pleasurable aspects of physical activity while discouraging solitary or goal-directed physical activity [57].

Recommendations from clinicians may also be utilized within the context of established treatment modalities. For example, several clinicians reported connecting physical activity with mindfulness or encouraging clients to engage in mindfulness practices in connection with physical activity (e.g., noticing their breathing during physical activity or scanning the body before and after physical activity). Mindfulness has its roots in ancient spiritual practice but has also been integrated into psychotherapeutic treatments across multiple evidence-based modalities (e.g., dialectical behaviour therapy, mindfulness-based cognitive therapy) [58]. Additionally, clinicians recommended physical activity within the framework of behavioural activation. Behavioural activation is a component of cognitive behaviour therapy for the treatment of depression [59]. It involves countering symptoms of depression by increasing adaptive activities through activity scheduling, goal setting, and self-monitoring [60]. Given that this is an existing, common treatment for depression, the use of this framework to recommend physical activity is unsurprising.

Several clinicians also discussed facilitating physical activity for their clients outside of the immediate psychotherapy session. For example, one practitioner worked in a multi-disciplinary setting and was able to refer clients directly to an exercise professional. Another coordinated their clients’ participation in an exercise class as part of a group therapy program. This was a fairly infrequent theme, and many practitioners may lack the resources to directly facilitate engagement in physical activity. However, structured programs with multidisciplinary support, including support from exercise professionals, have been successful when implemented in the community [61].

#### 4.1.2. Psychoeducation

When discussing physical activity with their clients, clinicians reported providing psychoeducation and intentionally framing discussions of physical activity. Education about mental health can be an effective component of treatment and has been found to increase adherence to treatment and improve outcomes [62,63]. When combined with physical activity, psychoeducation has been found to improve treatment adherence for individuals with severe mental disorders [64]. Several clinicians in our study noted that they discuss physical activity as part of educating their clients about mental health and effective treatment strategies. The framework through which this education is provided was also emphasized by clinicians. Specifically, they reported aiming to ensure that discussions promote enjoyable movement rather than promoting exercise as a means of weight loss. Higher levels of internalized weight stigma and appearance evaluation are associated with reduced enjoyment of physical activity and physical activity avoidance [65]. Preoccupation with body weight and shape is also a risk factor for the development of eating disorders [66]. Clinicians in our study seemed to be aware of these factors and strive to promote physical activity while remaining non-stigmatizing and avoiding the conflation of enjoyable physical activity with exercise for weight loss.

#### 4.1.3. In-Session Integration of Physical Activity

Clinicians discussed various ways they include physical activity directly within a therapy session. For example, some clinicians described engaging in ‘walk and talk therapy’, either in person or virtually during phone sessions. Walk and talk therapy is relatively new to empirical research, but there is some indication of its promise as a treatment tool [67,68]. Other clinicians reported including active games in group therapy, having active play resources available for play therapy, or using physical activity as tool for exposure to physical sensations associated with anxiety. Responses from clinicians indicate that there may be wide applicability of physical activity to a range of therapeutic techniques and modalities (e.g., cognitive behavioural therapy, play therapy, mindfulness-based therapies). Clinicians also described techniques for the use of physical activity in both individual and group therapy. The ability to integrate physical activity into sessions may be somewhat constrained by the clinical setting, but the clinicians in the current study saw it as a valuable addition to traditional talk therapy.

#### 4.1.4. Assessment of Physical Activity as an Alternative or Addition to Medication

The final two themes within the first research question included the assessment of physical activity and the discussion of physical activity as an alternative or addition to medication. Both of these themes were fairly infrequent. Some clinicians regularly ask about and assess their clients’ physical activity as part of treatment planning. Others provide education about physical activity as an alternative or addition to medication. Both themes suggest that clinicians are considering physical activity within the context of other aspects of client lifestyles and health.

### 4.2. How Do Clinicians Learn About Physical Activity and Mental Health?

The second research question addressed clinician education, training, and knowledge about the relationship between physical activity and mental health. Clinicians were asked where they had learned about the role of physical activity in mental health. Responses included personal experience, reading the literature, formal training opportunities, clinical experience, and consultation with colleagues. Interestingly, one of the most common responses was for clinicians to highlight the lack of training they received on this topic within their graduate education. Previous work has found that clinicians who are more knowledgeable about physical activity were more likely to prescribe it, but only a minority of mental health professionals could correctly identify the public health recommendations for adolescent physical activity [46]. This suggests that there may be a need for an increased emphasis on physical activity within educational programs for mental health professionals.

The clinicians in our study who were knowledgeable about physical activity reported self-directed learning. Several participants highlighted their own experience of physical activity and improvements in well-being. This is consistent with previous findings that clinicians’ own level of physical activity engagement predicts the frequency of physical activity prescription [37,69]. Other self-directed avenues for learning about physical activity included keeping up with the research literature, consulting with colleagues, and clinical experience. Some clinicians also had previous relevant training that was not part of their graduate program (e.g., undergraduate degree in kinesiology, yoga instructor training, nutrition training). This indicates that clinicians are employing a variety of avenues to obtain knowledge. However, this also raises the possibility that knowledge levels may vary significantly between clinicians. Given that clinician knowledge is a predictor of physical activity prescription [46], and a lack of knowledge is a barrier to physical activity prescription [42], increasing the overall knowledge level of clinicians may lead to increased integration of physical activity into mental health treatments. Graduate training programs may hold an opportunity to increase the prevalence of physical activity training for mental health professionals and reduce the knowledge discrepancies across clinicians.

### 4.3. What More Do Clinicians Want to Know About Physical Activity and Mental Health?

The clinicians in the current study also endorsed an interest in further training. Specifically, they reported an interest in resources for recommending physical activity, tailored to both professional and lay audiences. They also wanted more information about how to help their clients overcome barriers to physical activity participation. Clinicians seemed most interested in practical resources and tools that could be implemented directly with clients, but they tended not to report specific knowledge gaps. Evidence-based resources for healthcare providers to support individuals with mental health through physical activity do exist (e.g., The Exercise and Depression Toolkit) [70], but these resources may not be widely known by clinicians. Existing resources may not fully capture the needs of all populations, such as children or individuals from diverse cultural backgrounds. The development of additional resources, as well as increased efforts at dissemination of existing resources, may be beneficial for supporting clinicians to increase implementation of physical activity resources within their practice.

### 4.4. Limitations and Future Directions

This study contributes to our understanding of how physical activity is implemented in clinical mental health settings, and informs our knowledge of clinician training related to physical activity. However, there are some limitations to these findings. Firstly, the sample was self-selected, and participating clinicians likely had a greater interest in physical activity than clinicians who chose not to participate in the study. As such, this study may overestimate the extent to which a typical clinician is integrating physical activity into their psychotherapy practice. Additionality, the results rely on self-report data, which may be biased. Future research should recruit a larger sample with a more diverse group of clinicians to ensure that results reflect what is typical within the field as a whole. Clinicians in our study were all practicing in a single region (Ontario, Canada), which may further limit generalizability. Future research should additionally measure clinician knowledge directly to gain a better understanding of what clinicians know, and do not know, about physical activity and its role in mental health treatment, as this was lacking in the present study. The creation and evaluation of training resources and pilot programs to promote physical activity within a psychotherapeutic context would also provide valuable insight into what is feasible in this area.

## 5. Conclusions

The present study underscores clinician perspectives on the role of physical activity in mental health treatment and highlights their training in this area. Clinicians identified several strategies for implementing physical activity, including making client-specific recommendations, providing psychoeducation, in-session engagement in physical activity, and assessment of physical activity. They reported a range of sources of knowledge and training on this topic, but their exposure to physical activity within graduate education was limited. They also reported an interest in more training and resources that could be shared with their clients. Overall, clinicians valued physical activity as a therapeutic method and were interested in sharing the benefits of physical activity with their clients.

## Figures and Tables

**Table 1:** Strategies and processes to include physical activity (PA) in psychotherapy, identified by clinicians, by frequency of mention in interviews.

Clinician Strategies			Frequency
Making PA Recommendations			171
	Individualizing PA integration and recommendations		98
		Presenting issues	41
		Collaborative problem solving	32
	Mindful approach		28
	Behavioural activation		18
	Facilitating PA outside of practice		9
Psychoeducation			36
	Framing PA		17
In-session integration of PA			16
Asking as part of assessment/intake			9
Alternative/addition to medication			4

**Table 2:** Sources of information about physical activity (PA) and mental health identified by clinicians, by frequency of mention in interviews.

Sources of PA Knowledge	Frequency
Personal PA experience	18
Research/Literature	16
Lack of formal education	14
Formal training outside graduate program	8
Clinical experience	5
Colleague consultation	3

**Table 3:** Clinician-identified areas of future training and learning, by frequency of mention in interviews.

Clinician-Identified Training and Learning Needs	Frequency
Resources for recommending physical activity	35
Research summary for clinicians	15
Research summary for clients	13
Physical activity ideas	5
How to overcome barriers	7

## Data Availability

The data presented in this study are available on request from the corresponding author. The data are not publicly available due to privacy reasons given the interview-based nature of the data.

## Appendix A

Interview Questions

1. How do you use physical activity in your clinical practice? Provide an example.

2. Are there certain populations for whom you are most likely to recommend physical activity? Any populations where you would be less likely to recommend it?

3. Where have you learned about physical activity and mental health?

4. If you were to attend a training or access a professional resource on this topic, what information would you hope would be included?
